# Psychedelic concentrations of nitrous oxide reduce functional differentiation in frontoparietal and somatomotor cortical networks

**DOI:** 10.1038/s42003-023-05678-1

**Published:** 2023-12-19

**Authors:** Rui Dai, Zirui Huang, Tony E. Larkin, Vijay Tarnal, Paul Picton, Phillip E. Vlisides, Ellen Janke, Amy McKinney, Anthony G. Hudetz, Richard E. Harris, George A. Mashour

**Affiliations:** 1 Department of Anesthesiology, University of Michigan Medical School, Ann Arbor, MI 48109 USA; 2 Center for Consciousness Science, University of Michigan Medical School, Ann Arbor, MI 48109 USA; 3 Michigan Psychedelic Center, University of Michigan Medical School, Ann Arbor, MI 48109 USA; 4 Neuroscience Graduate Program, University of Michigan, Ann Arbor, MI 48109 USA; 5 Chronic Pain and Fatigue Research Center, University of Michigan Medical School, Ann Arbor, MI 48109 USA; 6 Department of Pharmacology, University of Michigan Medical School, Ann Arbor, MI 48109 USA

**Keywords:** Consciousness, Human behaviour

## Abstract

Despite the longstanding use of nitrous oxide and descriptions of its psychological effects more than a century ago, there is a paucity of neurobiological investigation of associated psychedelic experiences. We measure the brain’s functional geometry (through analysis of cortical gradients) and temporal dynamics (through analysis of co-activation patterns) using human resting-state functional magnetic resonance imaging data acquired before and during administration of 35% nitrous oxide. Both analyses demonstrate that nitrous oxide reduces functional differentiation in frontoparietal and somatomotor networks. Importantly, the subjective psychedelic experience induced by nitrous oxide is inversely correlated with the degree of functional differentiation. Thus, like classical psychedelics acting on serotonin receptors, nitrous oxide flattens the functional geometry of the cortex and disrupts temporal dynamics in association with psychoactive effects.

## Abstract (web summary)

Like classical psychedelics, nitrous oxide reduces functional differentiation in human frontoparietal and somatomotor networks, flattening functional brain geometry and disrupting temporal dynamics in association with the psychedelic experience.

## Introduction

As the scientific and clinical interest in psychedelic drugs continues to grow, there is a pressing need for a deeper neurobiological understanding. Nitrous oxide, an NMDA receptor antagonist^1^, has been in continuous clinical use as an anesthetic since the mid-19th century but at subanesthetic concentrations it has psychedelic effects^2^. Unlike classical serotonergic psychedelics such as lysergic acid diethylamide (LSD), psilocybin, and dimethyltryptamine (DMT)—nitrous oxide is inhaled as a gas, inducing profound alterations in consciousness within seconds. The unique characteristics of nitrous oxide result in vivid perceptual alterations, time distortion, and an extraordinary but short-lived psychedelic experience. Nitrous oxide is also being studied for therapeutic uses outside of anesthesia and analgesia, with recent investigations indicating efficacy as an antidepressant^3–5^.

Although there are extensive neuroimaging studies on the neural correlates of classical serotonergic psychedelics^6–8^, the neural underpinnings of the psychedelic experience induced by nitrous oxide have been relatively unexplored. This research gap endures despite the long history of this inhalational agent and the comprehensive description of its psychological effects by William James more than a century ago^9^. Although there have been several electroencephalography (EEG) and magnetoencephalography (MEG) studies that have shed light on its effects, the data primarily stem from investigations at sedative concentrations rather than psychedelic ones. These studies have characterized changes in spectral features, functional connectivity, and complexity during nitrous oxide exposure, offering valuable insights into its network-level mechanisms^10–15^.

To date, a single functional magnetic resonance imaging (fMRI) study^16^ has described functional alterations in brain networks linked to the psychedelic experience during nitrous oxide exposure. These changes manifest as a weakening of within-network functional connectivity coupled with a simultaneous strengthening of between-network functional connectivity, consistent with previous functional neuroimaging investigations into the acute effects of classical psychedelic drugs, such as LSD^17–19^. These observations indicate enhanced cross-talk among functionally distinct large-scale brain networks, reflecting an elevated level of functional integration or, alternatively, a reduced degree of functional differentiation. Nevertheless, the specific neural changes induced by various psychedelics, whether at the regional or network level, have exhibited limited convergence^20–27^.

A major gap in psychedelic neuroscience has been the limited comprehension of the brain’s overall spatiotemporal organization related to psychedelic effects. In terms of spatial organization, most investigations have been limited to describing neural correlates of psychedelics in terms of discrete brain regions or functional networks. In terms of temporal organization, research has been restricted to measuring neural activity through signal averaging. To advance the field, we investigated the connection between the spatiotemporal reconfiguration of brain activity and psychedelic effects induced by nitrous oxide.

Cortical gradient mapping stands as an innovative analytical tool for exploring the brain’s functional-spatial organization along a continuous spectrum^28–30^, distinguishing it from conventional techniques reliant on discrete boundaries, e.g., functional parcellation in neuroimaging. As an intuitive metaphor, consider defining a geographic region by its boundary coordinates, which is akin to functional parcellation, versus describing it by elevation slopes or changes in vegetation types across various topographical axes, which is similar to gradient mapping. These cortical gradients span a wide spectrum of functions and networks, ranging from perception and action to higher-order cognitive processes^28^. Notably, Gradient-1, known as the unimodal to transmodal gradient, enables the integration of sensory signals with non-sensory data, transforming them into abstract content. Gradient-2, the visual to somatomotor gradient, represents the specialization of different sensory modalities. Lastly, Gradient-3 spans functional distinctions ranging from regions typically deactivated during task performance (i.e., task-negative) to those activated in frontoparietal and attention networks (i.e., task-positive)^31,32^. Despite promising foundations, the potential of gradients as a framework for analyzing and conceptualizing non-ordinary states of consciousness induced by psychedelics remains ripe for exploration.

In addition to the brain’s functional geometry, dynamic processes continuously mold and reconfigure functional arrangements, leading to the evolution of brain activity patterns over time^33,34^. Recent empirical investigations have highlighted the intricate interplay between the spatial and temporal characteristics of brain activity, emphasizing that a comprehensive understanding necessitates the consideration of both aspects. Notably, transient fMRI co-activations^33,35,36^ spanning the entire cortex have been observed to propagate like waves, following the spatially defined cortical gradients^37–39^. Consequently, temporal dynamics are likely to be influenced by the underlying functional geometry. Exploring the co-variation between these spatial and temporal factors holds the potential to offer deeper insights into the neural underpinnings of psychedelic effects.

The objective of this study was to apply advanced cortical gradient mapping and co-activation pattern analysis to dissect the brain’s spatiotemporal reconfiguration during the psychedelic experience induced by nitrous oxide. Building upon previous research findings^16,25^, we tested the hypothesis that nitrous oxide could diminish functional differentiation within the human cortex, as evidenced by a contraction in functional geometry and a disruption in temporal dynamics. We reanalyzed a neuroimaging dataset of healthy human volunteers, who were assessed by fMRI before and during exposure to psychedelic concentrations of nitrous oxide (35%, in oxygen) and who completed a validated altered states of consciousness questionnaire^40^ before and after drug exposure. We quantified the changes of neural activity in cortical gradients and co-activations; we also performed correlation analyses to explore the relationship between subjective psychedelic experience and these brain measures. We demonstrate that nitrous oxide flattens the functional geometry of the cortex and disrupts related temporal dynamics, particularly within the frontoparietal and somatomotor networks, in association with the psychedelic experience.

## Results

### Nitrous oxide reduces functional differentiation in cortex

**Fig. 1:**
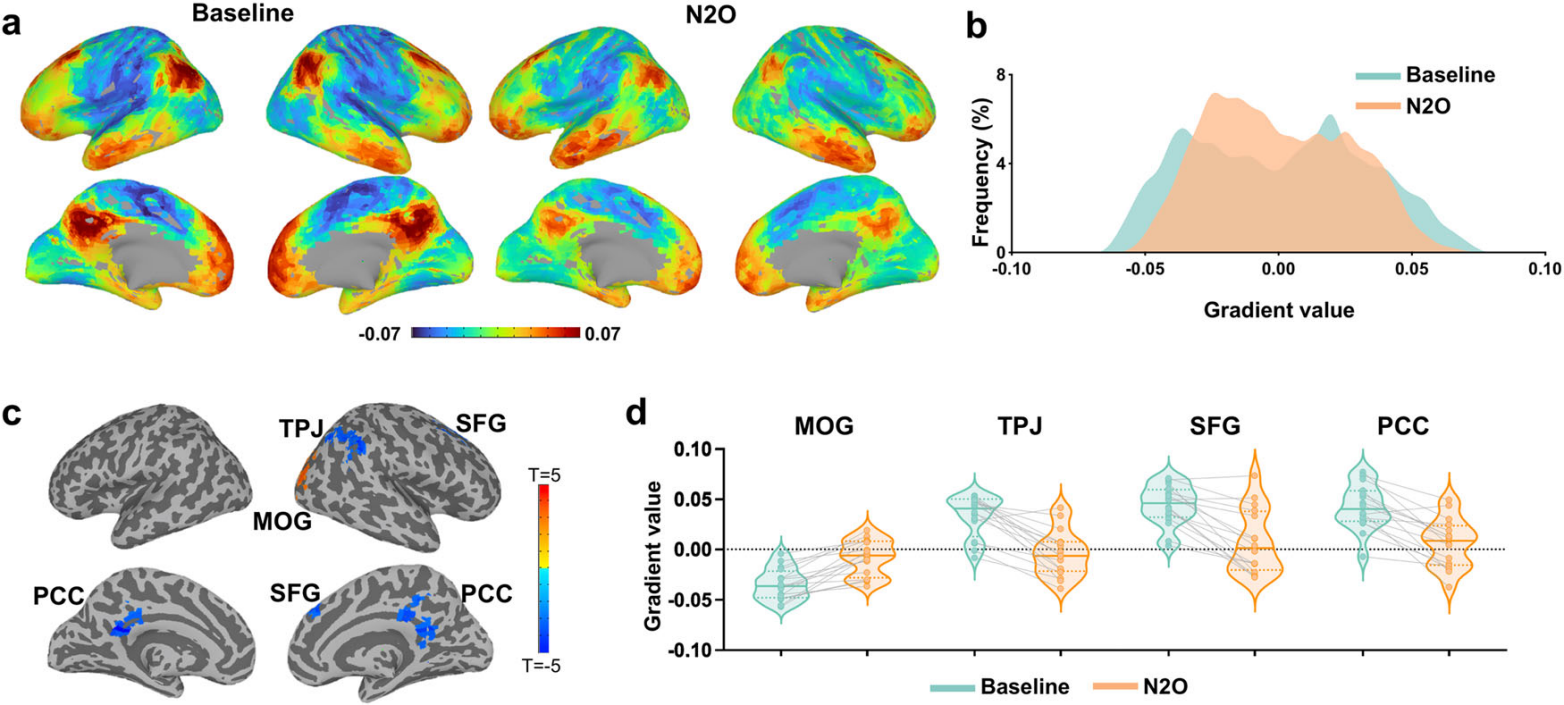
The principal cortical gradient during baseline and nitrous oxide. **a** Visualization of the principal gradient values of the cortex. The color scale represents gradient values, with warm colors indicating transmodal and cool colors indicating unimodal. **b** Histogram of principal gradient derived from whole-brain voxels. **c** Voxel-based contrast of the gradient values for nitrous oxide vs. baseline. Paired *t*-test (two-tailed) was performed for nitrous oxide condition (*n* = 16) against its own baseline conditions (*n* = 16), pFWE <0.05 corrected. **d** Individual gradient values extracted from regions showing statistical significance in (**c**). N_2_O nitrous oxide, MOG middle occipital gyrus, TPJ temporoparietal junction, SFG superior frontal gyrus, PCC posterior cingulate cortex.

In this study, we used cortical gradient analysis to investigate the functional brain connectome in a nonlinear diffusion space. We identified the major spatial axes of functional connectivity at the voxel level, based on the functional similarity structure of fMRI data (i.e., gradients). Voxels within each gradient are categorized according to their similarity in activity patterns, with those at one end of the gradient being more similar (less functionally differentiated) than those at the other. Our results showed that the principal gradient ranged from unimodal cortices (e.g., visual networks) to transmodal cortices (e.g., frontoparietal and default-mode networks) (Fig. 1a, b), which is consistent with previous studies^25,28,41,42^.

The voxel-based contrast map (nitrous oxide vs. baseline, pFWE <0.05 corrected) during nitrous oxide administration revealed a decrease of gradient values in the right lateral parietal/temporoparietal junction (TPJ), right superior frontal gyrus and bilateral posterior cingulate cortex (PCC), together with an increase of gradient values in the right middle occipital gyrus (Fig. 1c, d). In addition, we did not find a significant change of the overall range of the principal gradient (Fig. S1). Together, the results suggest that nitrous oxide induces a center-shifting of specific brain regions along the principal gradient, indicating a reduction of differentiation along the functional hierarchy.

**Fig. 2:**
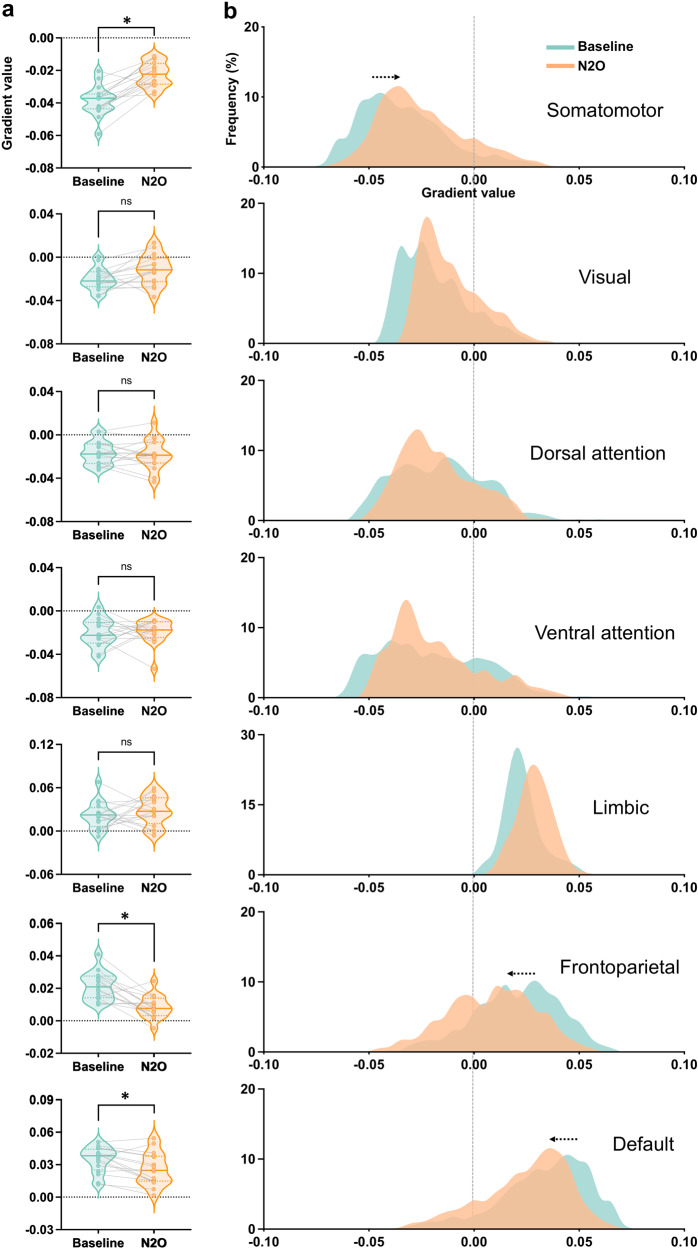
Principal gradient values in different functional networks. **a** Paired *t*-test of gradient values between nitrous oxide (*n* = 16) and baseline (*n* = 16) in seven networks. **b** histogram of gradient values in nitrous oxide and baseline in seven networks. * Bonferroni-corrected *p* < 0.05. N_2_O nitrous oxide.

We further quantified the cortical gradient changes at the network level. Using a seven-network parcellation scheme^43^, we found significant center-shifting during nitrous oxide administration in somatomotor (SMN; t_(15)_ = 6.86, *p* = 0.00003, Bonferroni-corrected), frontoparietal (FPN; t_(15)_ = 4.98, *p* = 0.001, Bonferroni-corrected), and default-mode (DMN; t_(15)_ = 3.48, *p* = 0.02, Bonferroni-corrected) networks (Fig. 2). Our network-based results indicated a specific reduction of differentiation at both the unimodal end (e.g., SMN) and transmodal end (e.g., FPN and DMN) along the functional hierarchy of the cortex during exposure to psychedelic concentrations of nitrous oxide.

Additionally, we conducted an analysis of the second and third-gradients of macroscale functional organization. The second gradient showed a range from visual to somatomotor cortices, and the third gradient showed a range from visual and default-mode areas to those commonly associated with multiple-demand tasks. However, we did not observe any significant changes during nitrous oxide administration in either gradient (Figs. S2 and S3).

### Nitrous oxide disrupts temporal dynamics

**Fig. 3:**
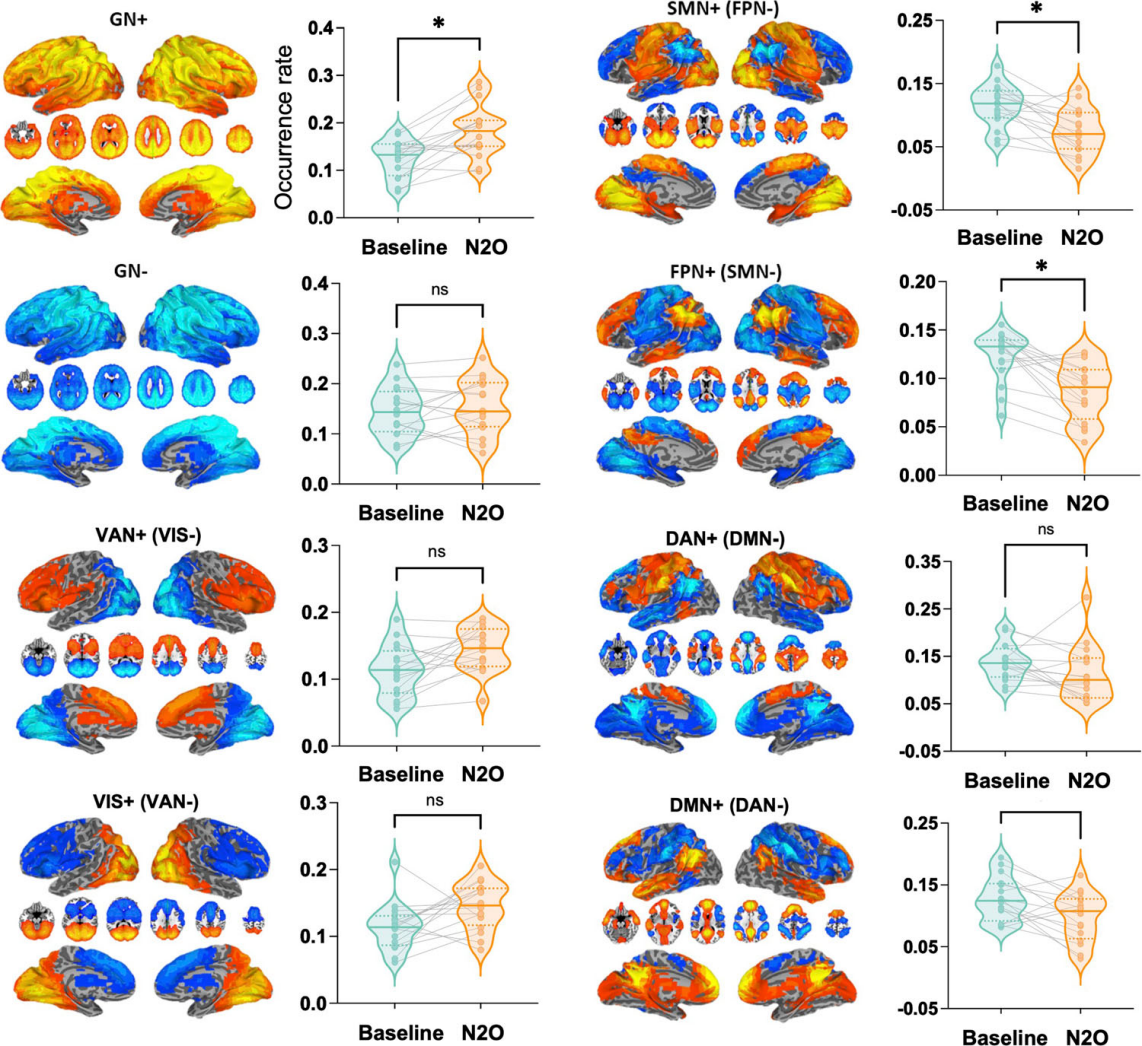
Co-activation patterns and their occurrence rates. Eight co-activation patterns are shown. Paired *t*-tests of the occurrence rates were performed for nitrous oxide (*n* = 16) vs. baseline (*n* = 16). * Bonferroni-corrected *p* < 0.05. N_2_O nitrous oxide, DMN+ default-mode network, DAT+ dorsal attention network, FPN+ frontoparietal network, SMN+ somatomotor network, VIS+ visual network, VAT+ ventral attention network, GN+ global network of activation, GN− global network of deactivation.

In order to examine the temporal evolution of brain activity, we conducted a co-activation pattern (CAP) analysis, which reflects transient covariations that form the basis for functional connectivity. Following our previous approach^33^, we identified eight CAPs, including default-mode network (DMN+), dorsal attention network (DAT+), frontoparietal network (FPN+), somatomotor network (SMN+), visual network (VIS+), ventral attention network (VAT+), and global network of activation and deactivation (GN+ and GN−). We calculated the occurrence rates of CAPs by dividing the number of fMRI volumes belonging to a given CAP by the total number of volumes per scan. We compared the occurrence rates between nitrous oxide and baseline in these eight CAPs (Fig. 3). Our results revealed a significant reduction in the occurrence rates of FPN+ (t_(15)_ = 4.79, *p* = 0.002, Bonferroni-corrected) and SMN+ (t_(15)_ = 3.94, *p* = 0.01, Bonferroni-corrected), accompanied by an increase in the occurrence rates of GN+ (t_(15)_ = 4.62, *p* = 0.003, Bonferroni-corrected) during nitrous oxide administration. The results indicate that FPN+ and SMN+ occurred less often during nitrous oxide administration, and the two CAPs seemed to be replaced by brain-wide co-activations (i.e., GN+). This is evidence in the temporal domain that nitrous oxide reduces the occurrence of differentiated functional CAPs involving the frontoparietal and somatomotor networks, while promoting a more globally integrated state.

In addition, we conducted Spearman correlation analyses to examine the relationship between cortical gradient values and occurrence rates of CAPs in the networks that exhibited significant results in both cortical gradient and CAP analyses (i.e., FPN and SMN). We observed a significant correlation between the FPN gradient score and FPN+ occurrence rate (Fig. S4), suggesting that the reduction of functional differentiation in the FPN along the cortical gradient was associated with a decrease in its occurrence rate.

### Spatiotemporal changes in functional networks are not attributable to head motion

First, we investigated whether differences in gradient values and CAP occurrence rates between nitrous oxide and baseline conditions were linked to discrepancies in head motion, quantified by average frame-wise displacements (FD). Our findings, detailed in Table S1 and Table S2, indicate a lack of statistically significant correlations, except for the limbic network. However, this correlation within the limbic network does not impact our primary results since gradient values in this network did not exhibit statistical significance when comparing nitrous oxide to baseline. Second, we performed ANCOVA analyses, incorporating FD as a covariate, to investigate the effect of nitrous oxide on gradient values and CAP occurrence rates (Table S3 and Table S4). The overall influence of FD was minimal, and our primary findings regarding the reduction in gradient values and alterations in co-activation due to nitrous oxide remained consistent. The only exception was the lack of significance in DMN gradient values when comparing nitrous oxide to the baseline, after accounting for FD. Third, we conducted a whole-brain principal cortical gradient contrast between nitrous oxide and baseline while accounting for FD as a covariate (Fig. S5). The resulting contrast map closely mirrors the findings presented in Fig. 1, providing further support that our results are not significantly influenced by head motion.

### Psychedelic phenomenology is linked to changes in cortical gradients and dynamic brain activity

**Fig. 4:**
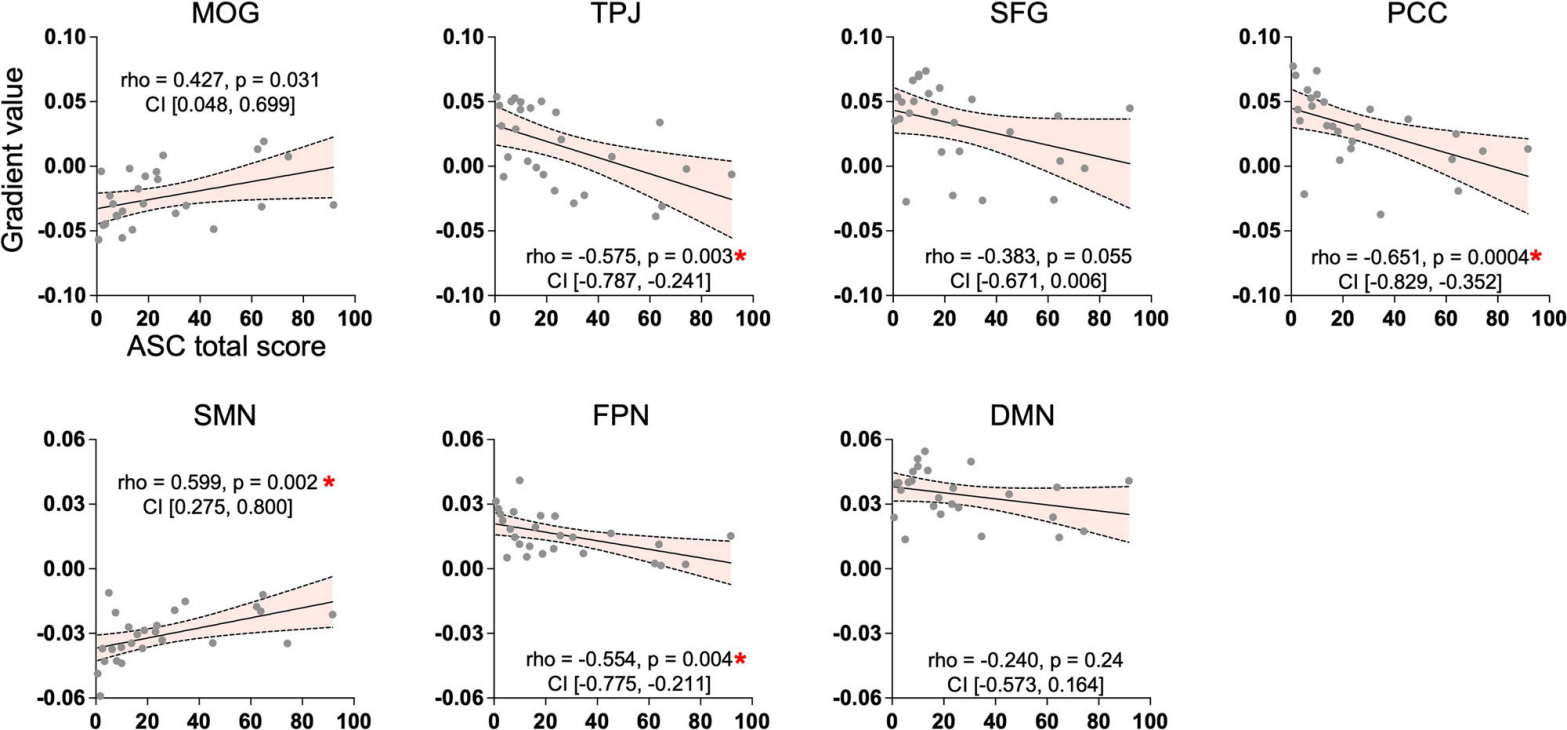
Spearman correlations between gradient values and the total score of 11D-altered states questionnaire. The total score in the questionnaire measures the intensity of psychedelic experiences, or the degree to which an individual’s conscious experience deviates from their ordinary state of consciousness during the psychedelic state. Higher scores indicate more intense experiences, while lower scores indicate milder effects. Spearman’s rank correlations were performed between gradient values and ASC total scores across 13 participants and two conditions (*n* = 26). Spearman’s rank correlation coefficient (rho), uncorrected *p* values, and 95% confidence interval (CI) are reported in each scatter plot. * Bonferroni-corrected *p* < 0.05. MOG middle occipital gyrus, TPJ temporoparietal junction, SFG superior frontal gyrus, PCC posterior cingulate cortex, SMN somatomotor network, FPN frontoparietal network, DMN default-mode network.

To test associations between the degree of changes in cortical gradients and dynamic brain activity with the subjective intensity of the psychedelic state induced by nitrous oxide, we performed correlation analyses between the principal gradient values and the total score derived from altered-states-of-consciousness (11D-ASC) questionnaire (see Fig. S6 for questionnaire statistics). The analyses involved four regions (middle occipital gyrus, superior frontal gyrus, TPJ, and PCC) identified during voxel-level analysis as well as three networks (SMN, FPN, DMN) identified during network-level analysis. Note that these regions or networks were determined independently of behavioral assessment. Our results revealed significant correlations between altered states of consciousness and the degree of reduction of functional differentiation in TPJ, PCC, SMN, and FPN (Bonferroni*-*corrected *p* < 0.05, Fig. 4). These findings suggest that the degree of subjective intensity of the psychedelic state induced by nitrous oxide was associated with changes in cortical network gradients. Additionally, we conducted exploratory voxel-based analyses across the entire brain without constraining the analysis to specific regions or networks. These results reaffirmed our initial findings, with significant correlations observed primarily in regions such as the TPJ, PCC, and within SMN and FPN (Fig. S7).

**Fig. 5:**
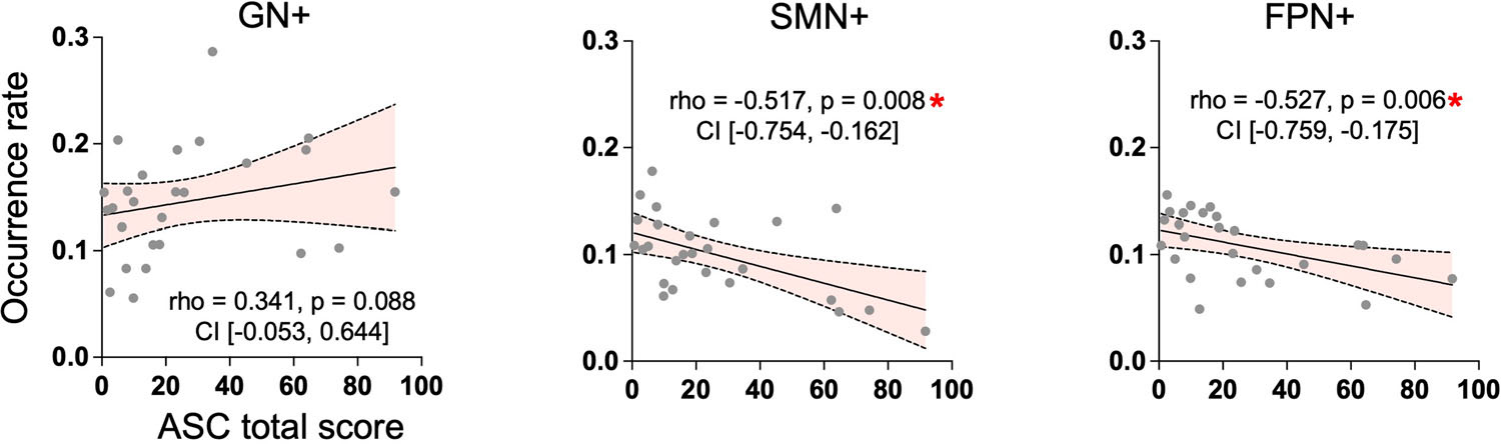
Spearman correlations between gradient values and occurrence rates. Spearman’s rank correlations were performed between occurrence rates and altered states of consciousness questionnaire total scores across 13 participants and two conditions (*n* = 26). Spearman’s rank correlation coefficient (rho), uncorrected *p* values, and 95% confidence interval (CI) are reported in each scatter plot. * Bonferroni-corrected *p* < 0.05. SMN+ somatomotor network, FPN+ frontoparietal network. GN+ global network of activation.

We also conducted correlation analyses between the occurrence rates of CAPs and the total score of the altered states of consciousness questionnaire in three networks that displayed statistical significance in the CAP analysis (GN+, SMN+, FPN+). We observed a significant negative correlation in both SMN+ and FPN+ (Fig. 5), suggesting that the less frequently these two CAPs occurred, the more intense the psychedelic experience was.

We further explored the 11 dimensions of the 11D-ASC, conducting additional correlation analyses between gradient values, CAP occurrence rates, and each dimension (Table S5). Our findings build upon our earlier observations regarding the associations between the total questionnaire score and gradient values or CAP occurrence rates within the SMN and FPN. Notably, these same networks also displayed significant correlations with individual altered states of consciousness dimensions, offering a more detailed understanding of how specific facets of the psychedelic experience relate to neural changes. However, it is important to exercise caution in interpretation due to the limited sample size and the potential for false positives resulting from the numerous correlation analyses conducted.

## Discussion

We present evidence that psychedelic concentrations of nitrous oxide induce specific functional reorganizations in the brain, including a reduction of functional differentiation in four brain regions (TPJ, PCC, superior frontal gyrus, and middle occipital gyrus) and three networks (SMN, FPN, and DMN) that are distributed along the hierarchical extremes of the principal/first cortical gradient. Co-activation pattern (CAP) analysis showed a reduced occurrence rate of frontoparietal and somatomotor networks and an enhanced occurrence rate of global co-activation. Finally, we found correlations between the psychedelic experience induced by nitrous oxide and this particular spatiotemporal reorganization of brain activity. In summary, our data suggest that nitrous oxide flattens the functional geometry of the cortex and disrupts the related temporal dynamics, particularly in frontoparietal and somatomotor networks, in association with psychedelic experience.

Although most neuroimaging studies have focused on the neural correlates of classical psychedelics such as LSD, psilocybin, and DMT, our study is among the few that has investigated nitrous oxide. We utilized two advanced approaches, cortical gradient, and CAP analyses, to provide spatially/regionally specific evidence about the neural correlates of the psychedelic state induced by nitrous oxide. A main finding is the reduced functional differentiation in both functional geometry and temporal dynamics, which is aligned with prior research demonstrating that psychedelics reduce functional integration within networks and enhance functional integration between networks^16–19^. More specifically, our study revealed reduced differentiation in the frontoparietal and somatomotor networks along the unimodal-transmodal axis of functional connectivity similarity, with reduced occurrence rates in temporal dynamics. These networks were increasingly replaced by global co-active networks.

Reduced functional differentiation denotes a decreased distinction or separation across brain functions. This means that, under the influence of nitrous oxide, certain brain regions or networks that typically exhibit distinct patterns of activity may become less distinct from each other in terms of their functional connectivity profiles. The finding that frontoparietal and somatomotor networks are most affected by nitrous oxide raises intriguing questions about the drug’s neurobiological mechanisms and its unique subjective effects. One possibility is that these networks are particularly sensitive to the effects of nitrous oxide due to their integrative role in cognitive and somatomotor function. The frontoparietal network is associated with higher-order cognitive functions, and the somatomotor network plays a fundamental role in motor control^43–46^. Further research is needed to identify the reasons why nitrous oxide differentially affects these particular networks, which could include distribution of molecular targets for the gas or more of a systems-level phenomenon.

Our study may contribute to the development of mechanistic models of nitrous oxide by providing insights into how the drug affects large-scale brain networks. Although prior studies have focused primarily on EEG or MEG, our fMRI findings offer a complementary perspective. In particular, studies have highlighted the impact of nitrous oxide on brain oscillatory power, EEG/MEG topography, and functional connectivity, setting it apart from other anesthetic agents^10–12,14^. Furthermore, low-dose nitrous oxide has been linked to psychometric impairment and changes in EEG complexity, particularly in areas associated with cognitive function^15^. Notably, a previous EEG study explored the effects of nitrous oxide-induced sedation on frontal-parietal functional connectivity^13^. Nitrous oxide led to increased low-frequency activity in frontal regions, affecting feedback and feedforward connections differently across frequency bands. Although our study cannot directly correlate with prior EEG/MEG data, it contributes valuable insights into the impact of nitrous oxide on reshaping the functional network geometry and large-scale temporal dynamics of the brain.

Our findings are consistent with previous research demonstrating altered functional connectivity in these networks with classical psychedelics. For example, previous studies of LSD have shown increased global functional connectivity in regions spanning the default-mode and frontoparietal networks^25,26^ as well as in somatomotor and visual networks^24,27^. Notably, a recent study demonstrated that psilocybin and LSD administration significantly reduces cortical gradients, disrupting brain region hierarchy^25^. These psychedelics increase connectivity between the transmodal cortex and the rest of the brain, including unimodal areas, and disrupt intra-unimodal functional connectivity. Our study extends these findings to nitrous oxide-induced psychedelia. Specifically, we observed that as the gradient in the SMN decreased, the occurrence rate of whole-brain global co-activation increased, while the exclusive occurrence rate within the SMN decreased. These results are consistent with heightened communication across the entire brain and disruption of intra-unimodal functional connectivity. In our previous nitrous oxide fMRI study^16^, we reported similar increases in inter-network connectivity and decreases in intra-network connectivity. These findings collectively suggest that psychedelics with various molecular mechanisms reconfigure brain organization by enhancing connectivity between diverse networks while reducing connectivity within specific networks.

Also of relevance, a previous study showed that LSD and psilocybin can flatten the brain’s control energy landscape, which reduces energetic barriers and enables the brain to more easily navigate its repertoire through state transitions^34^. In other words, brain states are less “sticky” and can more flexibly move from one state to another^18^. Our results on nitrous oxide indicate that the flattened energy landscape may be associated, in part, with de-differentiation of executive control (frontoparietal) and motor control (somatomotor) networks, promoting greater integration and communication between these control systems and other brain areas. However, it is still unclear how a change of functional differentiation along cortical gradients relates to a change of brain entropy^47,48^, metastability^49–51^, or dynamic repertoires^52^.

We also observed a reduction of functional differentiation in the TPJ and PCC. This finding is in agreement with previous studies that have reported increased global connectivity in these regions due to LSD^26^ and increased connectivity between the right TPJ and other regions of the posterior cortex during exposure to nitrous oxide, ketamine, and LSD^16^. Together, our results suggest that the modulation of these regions may contribute to the phenomenology of the psychedelic experience, which is supported by past high-density EEG studies of ketamine^53^.

There are numerous limitations to this investigation. First, our results were primarily based on the cortex; future investigation of subcortical regions using high-field MRI will be important to clarify fully the neurobiology of the psychedelic state induced by nitrous oxide. Second, the present study examined the network-level effects of nitrous oxide only, highlighting the need for further comparison with other psychoactive drugs that have not been studied using these techniques, such as DMT and methylenedioxymethamphetamine. Third, the sample size of our study was small and may not be fully representative of the general population. Despite these limitations, this study is the first to characterize cortical gradient and temporal dynamic changes during the administration of psychedelic concentrations of nitrous oxide. The present study extends previous findings on the neural underpinning of the psychedelic state induced by 5-HT2 modulators and provides novel neural correlates of altered subjective experiences induced by nitrous oxide.

## Methods

The study was performed at the University of Michigan Medical School and received approval from the Institutional Review Board under the identifier HUM00096321. Prior to participation, all subjects were carefully informed about the potential risks and benefits of the study, and written informed consent was obtained from each individual. The analysis of this study was part of a clinical trial that was registered with clinicaltrials.gov under the identifier NCT03435055, and the primary study’s results were released in July 2021. There were two adverse events reported, one requiring pharmacological treatment for nausea; in both cases, the experiment was terminated and the neuroimaging results were not included in subsequent analysis.

### Participants

In this study, sixteen healthy participants (8 males, means ± SD, ages: 24.6 ± 3.7 years) underwent two resting-state fMRI scans before and during exposure to subanesthetic levels of nitrous oxide (i.e., 35% concentration). Two participants were excluded because of excessive head motion (more than a half TRs in each scan). Among these sixteen participants, thirteen produced complete data from the altered states questionnaire, whereas three did not complete the questionnaire. Requirements for participation, included being classified as physical status I by the American Society of Anesthesiologists, being free of drug abuse or psychosis, and being free of other health-related conditions (https://www.clinicaltrials.gov/ct2/show/NCT03435055).

### Experimental design

The study consisted of two visits for participants; a pre-scan visit and a scanning visit within three days. During the pre-scan visit, participants were informed of the study protocol. During the scanning visit, participants underwent fMRI data collection during both placebo and subanesthetic nitrous oxide inhalation. Prior to the resting-state scan, nitrous oxide was administered to achieve at least 5 min of equilibrium, and any adverse physiological or psychological reactions were monitored and addressed. Before scanning and after 30 min of recovery from nitrous oxide administration, the altered states consciousness questionnaire^40^ was administered.

### Drug administration

The administration of nitrous oxide was conducted using MRI-compatible anesthesia machines, overseen by at least two fully trained anesthesiologists. Prior to imaging, nitrous oxide was first administered outside of the scanner to ensure airway patency and physiological stability. To mitigate predicable common side effects, participants were given ondansetron (4–8 mg IV) with an additional dose of dexamethasone (4 mg IV) if necessary; glycopyrrolate (0.2–0.4 mg IV), labetalol (5–10 mg/kg IV), and midazolam (1–2 mg IV) were available if necessary.

Standard intraoperative monitoring devices, including electrocardiogram, blood pressure, pulse oximetry, and capnography, were used throughout the experiment. To reduce interference from external stimuli, participants wore earplugs and headphones during the fMRI scanning.

### fMRI data acquisition

Imaging data were obtained using a 3 T Philips Achieva MRI scanner (Best, Netherlands) located at Michigan Medicine, University of Michigan. Functional whole-brain images were acquired using a T2*-weighted echo-planar sequence with the following parameters: 48 slices, TR/TE = 2000/30 ms, slice thickness = 3 mm, field of view = 200 × 200 mm, flip angle = 90˚, and scan time of 6 min. High-resolution anatomical images were also acquired for coregistration with the resting-state fMRI data.

### Altered states questionnaire

There are 11 dimensions included in the altered states of consciousness questionnaire, including the following: experiences of unity, spiritual experience, blissful state, insightfulness, disembodiment, impaired control and cognition, anxiety, complex imagery, elementary imagery, audiovisual synesthesia, and changed meaning of percepts. Participants were asked to rate their experiences on each dimension using a discrete response scale with 11 options, ranging from 0 (Never) to 10 (Always). The reported scale scores were calculated by averaging the responses across all items belonging to each respective scale. Thirteen out of the 16 participants completed the survey.

### fMRI data preprocessing

The fMRI data preprocessing steps were conducted using AFNI (http://afni.nimh.nih.gov/). The following procedures were applied: (1) Removal of the first two frames of each scan; (2) Slice timing correction; (3) Rigid head motion correction/realignment. The frame-wise displacement (FD) of head motion was calculated as the Euclidean Norm of the six-dimension motion derivatives. Any frame with a derivative value exceeding the FD of 0.4 mm was removed, along with its previous frame; (4) Coregistration with T1 anatomical images; (5) Spatial normalization into Talaraich stereotactic space and resampling to 4 mm isotropic voxels; (6) Time-censored data was band-pass filtered to 0.01–0.1 Hz using AFNI’s function 3dTproject. Linear regression was used to remove undesired components such as linear and nonlinear drift, time series of head motion and its temporal derivative, and mean time series from the white matter and cerebrospinal fluid; (7) Spatial smoothing with a 6 mm full-width at half-maximum isotropic Gaussian kernel; (8) Normalization of each voxel’s time series to zero mean and unit variance.

### Cortical gradient analysis

Cortical gradient analysis was performed at the voxel level. The fMRI time series were first extracted from each voxel, and a 14871 × 14871 connectivity matrix was constructed for each participant and condition using Pearson correlation. The BrainSpace toolbox (https://brainspace.readthedocs.io/en/latest/) implemented in MATLAB R2022a was utilized for conducting cortical gradients analysis^54^. Based on previous work^28,38,55^, the connectivity matrix was first z-transformed and thresholded at a sparsity of 90%. This resulted in only the top 10% of weighted connections per row being retained. Subsequently, a normalized cosine angle affinity matrix was calculated to measure the similarity of connectivity profiles between different cortical areas. Based on a diffusion map embedding algorithm, gradient components were identified, which estimated the low-dimensional embedding from the high-dimensional connectivity matrix. The parameter α determines the degree to which the density of sampling points on the manifold influences the algorithm, where α = 0 signifies maximal influence and α = 1 indicates no influence. Additionally, the parameter t controls the scale of eigenvalues of the diffusion operator. Following recommendations of previous studies^28,41,54–56^, we fixed global relations between data points in the embedded space by setting α to 0.5 and t to 0, which indicated that the estimation of diffusion time is automated and derived through a damped regularization process^28,54^. It is important to highlight that our choice of key parameters, sparsity, and α, was guided by insights gleaned from one of our previous studies^42^. This prior research demonstrated clearly that opting for a sparsity threshold of 90% was not only conducive but optimal for effectively detecting group disparities. Additionally, the parameter α was found to have minimal impact on the overall robustness of the results.

The gradient solutions were aligned to a subsample of the human connectome project dataset (*n* = 100) using Procrustes rotation. This method involves finding an orthogonal linear transformation that superimposes a given source S onto a target T representation, effectively aligning the two representations^57^. The Procrustes rotation transformation was implemented to address the issue of eigenvector multiplicity and sign ambiguity, which may cause the computed gradients from different individuals to be incomparable. The alignment step improves the stability of gradient estimation and enables better comparability with existing literature^54^. The gradient eigenvector loading values from seven pre-defined functional networks^43^ were used to depict the cortical gradient organization at the network level. Note that the statistical analysis was conducted using individual cortical gradient values, which were computed based on individual connectivity matrices. The cortical gradients generated from group-average connectivity matrices were utilized primarily for visualization purposes.

### Tracking large-scale co-activation patterns

The brain is in a constant state of flux, continually altering its functional connections and evolving over time. Rather than averaging activity over long periods, we employed a supervised co-activation pattern (CAP) analysis. This method allows us to categorize dynamic BOLD intensity maps, obtained at every 2-second time point, into distinct brain states or pre-determined CAPs. This methodology extends beyond merely identifying similar BOLD fluctuations between individual voxels; it is a means of unveiling how distinct brain regions collaborate at a specific moment. The key steps in this process are as follows.

1. *How CAPs were determined*. The centroids of CAPs were originally determined through an unsupervised machine learning approach utilizing the k-means clustering algorithm in a large sample of fMRI data^33^. This algorithm categorizes a set of objects, in our case, fMRI volumes, into distinct groups or patterns. Each fMRI volume was the BOLD intensity map obtained at each time point. The objective of this k-means clustering algorithm is to minimize differences within each category while maximizing differences between them. These centroids represented various functional networks, including the default-mode network (DMN+), dorsal attention network (DAN+), frontoparietal network (FPN+), somatomotor network (SMN+), visual network (VIS+), ventral attention network (VAN+), and global network of activation and deactivation (GN+ and GN−). These eight CAPs were organized into four pairs of “mirror” motifs, each exhibiting strong negative spatial similarity^33^. For instance, DMN+ (i.e., activation) is associated with co-deactivation of the dorsal attention network (DAN-) while, conversely, DAN+ is associated with co-deactivation of the default-mode network (DMN−).
2. *Measuring CAP similarities*. At each 2-second time point (given the fMRI data acquisition has a repetition time of 2000 ms), we aimed to determine which predefined CAP best represents the brain’s activity. To achieve this, we assessed how closely the actual brain activity aligns with each predefined CAP centroid using Pearson’s correlation coefficient. In essence, we calculated how similar the brain’s activation pattern at a specific time point (represented as a vector of 14871 BOLD intensity values) was to each predefined CAP centroid (also represented as a vector of 14871 values).
3. *Assigning CAP labels*. Based on the degree of similarity calculated in the previous step, we assigned a CAP label to the brain activity at that specific time point. The CAP label corresponded to the predefined CAP centroid to which the brain activity was most similar.
4. *Quantifying occurrence rates*. We calculated the occurrence rate of each CAP during a specific time period (e.g., one resting scan) for each individual. This rate reveals how often a particular functional network was active during a specific state, such as under the influence of nitrous oxide or during normal wakefulness.

### Statistics and reproducibility

To correct for multiple comparisons in voxel-based gradient analysis, Monte Carlo simulation was used via the AFNI program 3dClustSim, resulting in a family-wise error rate of *p* < 0.05 with a minimum cluster size of 60 voxels. For network-based gradient analysis (mean gradient values) and CAP analysis, paired *t*-tests were conducted comparing the nitrous oxide condition (*n* = 16) to the baseline (*n* = 16). The Spearman correlations were used to analyze the relationship between the altered states questionnaire total scores and gradient values, as well as between the altered states questionnaire total scores and CAP occurrence rates, across 13 participants and two conditions (*n* = 26). Bonferroni correction, i.e., dividing the critical *P* value α = 0.05 by the number of comparisons being made, was used to counteract the multiple comparisons problem. JASP (v0.16.3; https://jasp-stats.org/) was used for statistical calculations.

### Reporting summary

Further information on research design is available in the Nature Portfolio Reporting Summary linked to this article.

### Supplementary information

Supplementary Information

Description of Additional Supplementary Files

Supplementary Data 1

Reporting Summary

## Data Availability

All data supporting the findings of this study are provided in Supplementary Data 1. Access to additional data are not openly available due to reasons of participant privacy and are available from the corresponding author upon reasonable request. Data are located in controlled access data storage at University of Michigan Medical School.

## Code availability

Publicly available software and toolbox used for analyses include AFNI (https://afni.nimh.nih.gov/), JASP (v0.16.3; https://jasp-stats.org/), and the Matlab BrainSpace toolbox (https://brainspace.readthedocs.io/en/latest/).
