# Supplementary material for: Psychedelic concentrations of nitrous oxide reduce functional differentiation in frontoparietal and somatomotor cortical networks
Source: Commun Biol. 2023 Dec 19;6:1284. doi: 10.1038/s42003-023-05678-1 (PMC10730842; doi:10.1038/s42003-023-05678-1)
Supplement: Supplementary file 1 — Supplementary Information [file 42003_2023_5678_MOESM1_ESM.pdf]

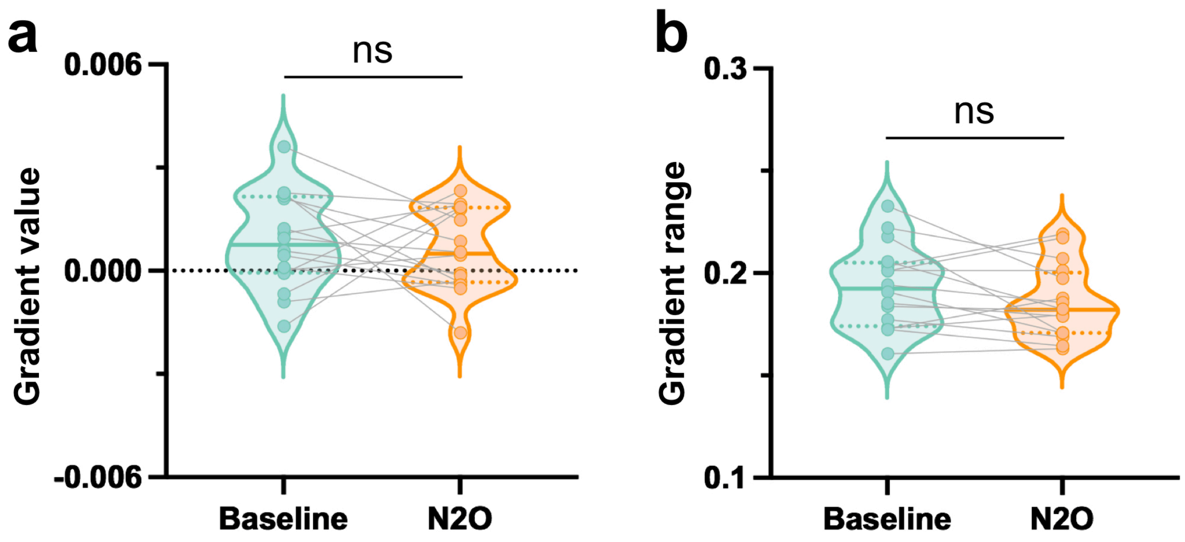

Figure S1: Principal functional cortical gradient value and range during nitrous oxide and baseline. **a** Paired t-test of the cortex-wide mean gradient value between nitrous oxide (n=16) and baseline (n=16). **b** Paired t-test of the gradient range between nitrous oxide (n=16) and baseline (n=16). Numerical range of each gradient was calculated as the distance from the minimum to the maximum gradient eigenvector values, indicating segregation (i.e., different connectivity profile) of the gradient extremes. N2O: nitrous oxide.

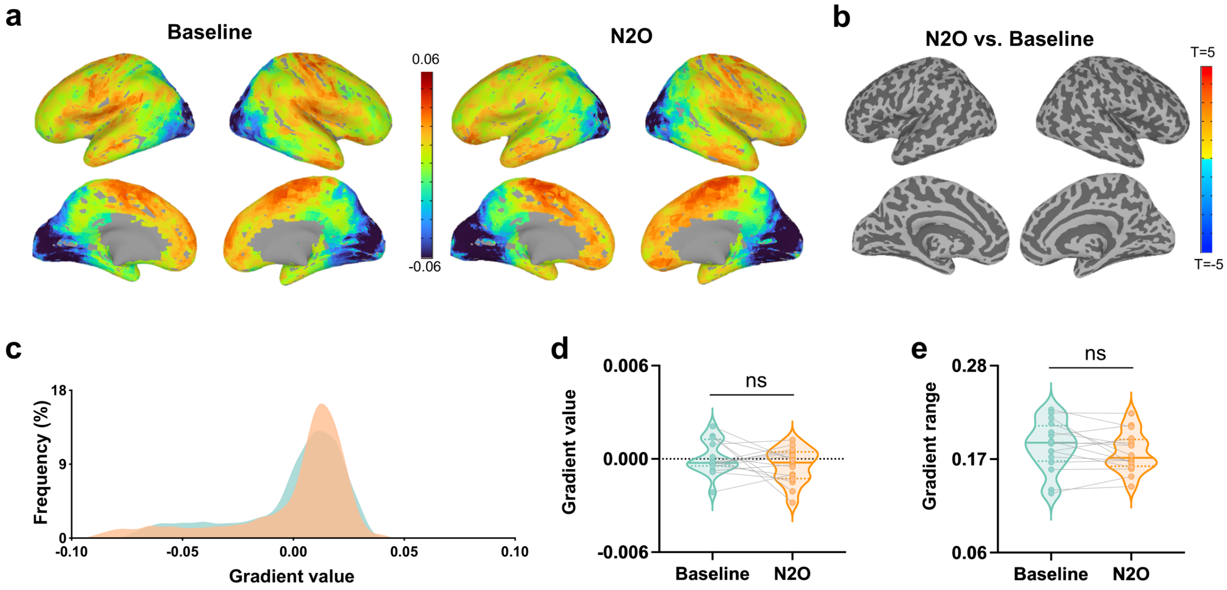

Figure S2: Secondary functional cortical gradient during nitrous oxide and baseline. **a** Global gradient values from visual to somatomotor axis. **b** Voxel-based contrast of the gradient values by nitrous oxide (n=16) vs. baseline (n=16). **c** Global histogram of the secondary gradient. **d** Paired t-test of the gradient value between nitrous oxide (n=16) and baseline (n=16). **e** Paired t-test of the gradient range between nitrous oxide (n=16) and baseline (n=16). N2O: nitrous oxide.

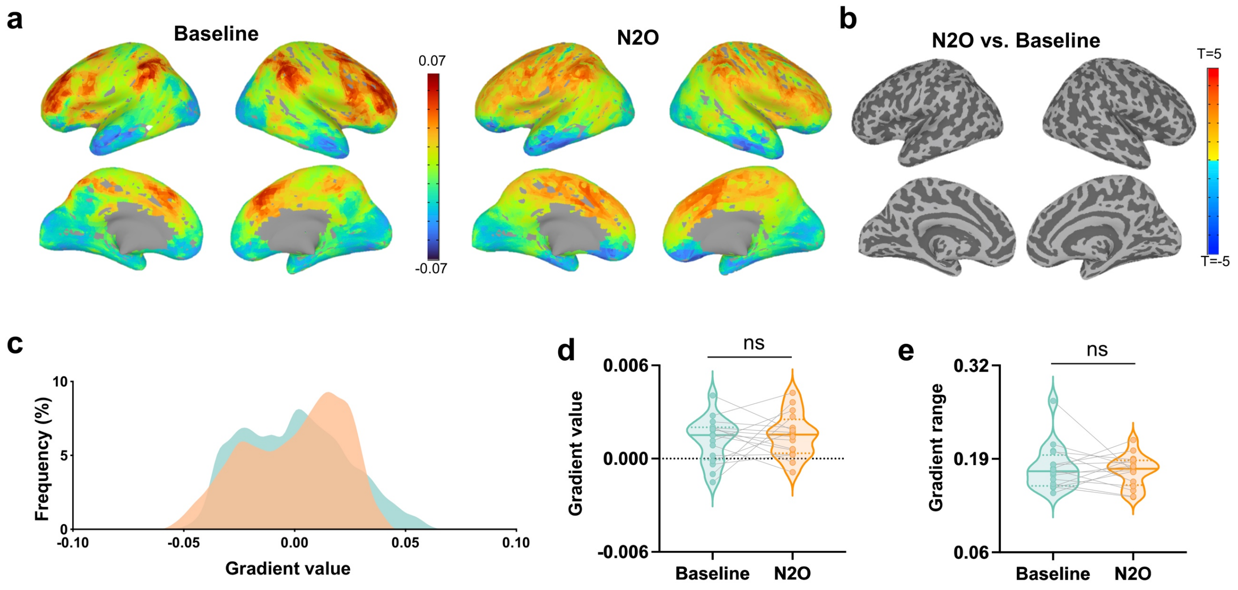

Figure S3: Third functional cortical gradient during nitrous oxide and baseline. **a** Global gradient values from visual to default-mode axis. **b** Voxel-based contrast of the gradient values by nitrous oxide (n=16) vs. baseline (n=16). **c** Global histogram of the third gradient. **d** Paired t-test of the gradient value between nitrous oxide (n=16) and baseline (n=16). **e** Paired t-test of the gradient range between nitrous oxide (n=16) and baseline (n=16). N2O: nitrous oxide.

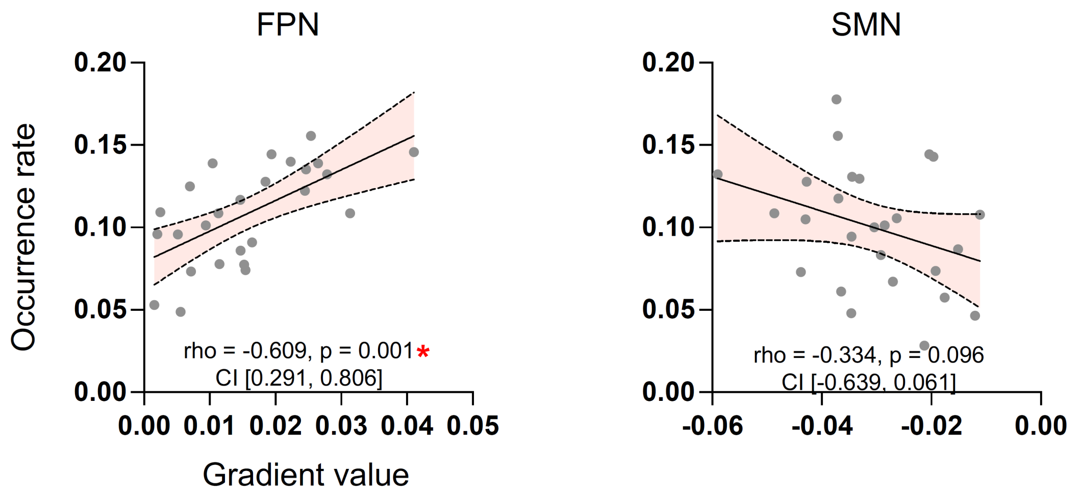

Figure S4: Spearman correlations between gradient score and occurrence rate. Spearman's rank correlations were performed between occurrence rates and gradient values across 13 participants and two conditions (n=26). Spearman's rank correlation coefficient (rho), uncorrected p values, and 95% confidence interval (CI) are reported in each scatter plot. * Bonferroni-corrected $p < 0.05$. FPN: frontoparietal network. SMN: somatomotor network.

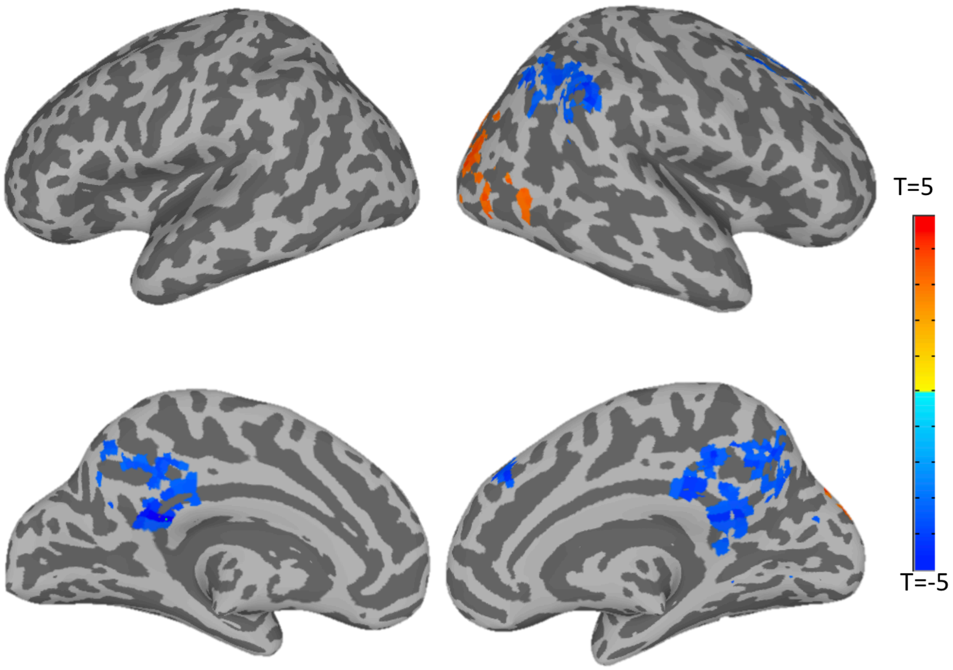

Figure S5: Voxel-based principal cortical gradient contrast map with frame-wise displacement (FD) as a covariate. We conducted a whole-brain principal cortical gradient contrast between nitrous oxide (n=16) and baseline (n=16) ($p < 0.05$, corrected) while accounting for FD as a covariate.

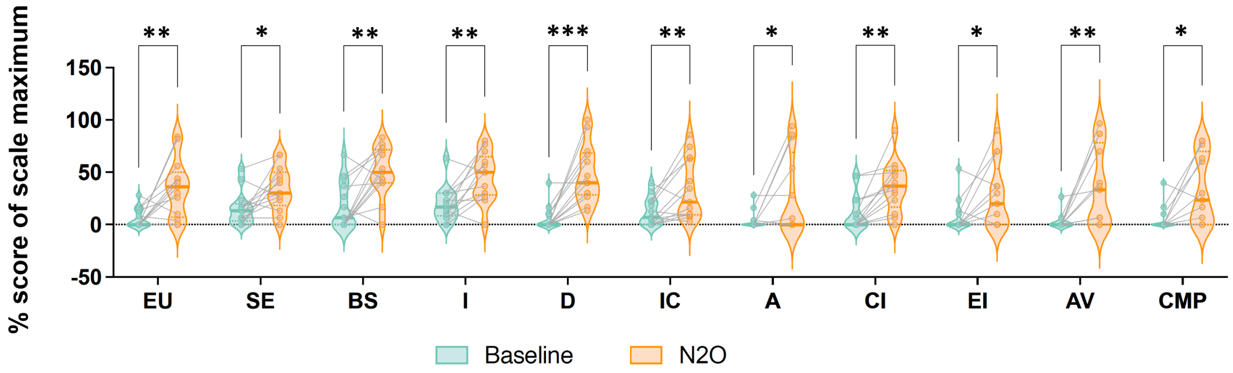

Figure S6: Behavioral results derived from the 11D-altered states questionnaire. Nitrous oxide (N2O) administration resulted in significantly higher total scores (n=13) than that of baseline condition (n=13) (mean ± SEM, baseline: 10.01 ± 2.88, nitrous oxide: 37.12 ± 6.69, $t(12) = 3.86$, $p = 0.002$). Subscales include the following, EU: experience of unity, SE: spiritual experience, BS: blissful state, I: insightfulness, D: disembodiment, IC: impaired control and cognition, A: anxiety, CI: complex imagery, EI: elementary imagery, AV: audio-visual synesthesia, CMP: changed meaning of percepts. * $p < 0.05$, ** $p < 0.01$, *** $p < 0.001$.

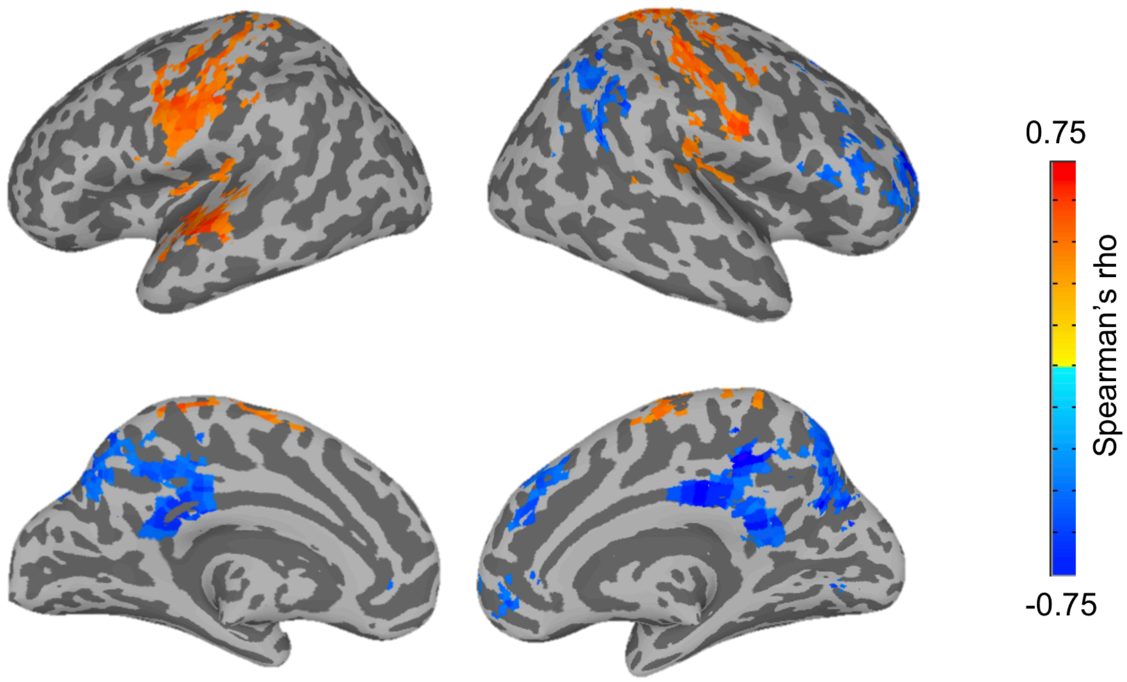

Figure S7: Exploratory whole-brain correlation analyses between the gradient values and Altered States of Consciousness questionnaire total score. Spearman correlation was performed on the voxel-based principal cortical gradient maps (across all subjects, $p < 0.05$, uncorrected) and the total scores across 13 participants and two conditions (n=26).

Table S1: Correlations between frame-wise displacement and the principle gradient values

			Pearson's r	p	p(B)
FD	-	SMN	-0.119	0.661	1
FD	-	VIS	0.271	0.309	1
FD	-	DAN	-0.084	0.757	1
FD	-	VAN	0.027	0.922	1
FD	-	LIM	0.645	0.007	0.049
FD	-	FPN	-0.446	0.084	0.588
FD	-	DMN	-0.579	0.019	0.133

Pearson correlations were performed between differences in gradient values (n=16) between nitrous oxide and baseline conditions and discrepancies in head motion quantified by average frame-wise displacements (n=16). FD: frame-wise displacement, SMN: somatomotor network, VIS: visual network, DAN: dorsal attention network, VAN: ventral attention network, LIM: limbic network, FPN: frontoparietal network, DMN: default-mode network, p(B): Bonferroni–corrected p.

Table S2: Correlations between frame-wise displacement and co-activation pattern occurrence rates

			**Pearson's r**	**p**	**p(B)**
FD	-	GN+	0.255	0.341	1
FD	-	GN-	0.285	0.284	1
FD	-	VAN+	0.488	0.055	0.44
FD	-	VIS+	0.201	0.456	1
FD	-	SMN+	-0.393	0.132	1
FD	-	FPN+	-0.247	0.356	1
FD	-	DAN+	-0.316	0.233	1
FD	-	DMN+	-0.349	0.185	1

Pearson correlations were performed between differences in co-activation pattern occurrence rates (n=16) between nitrous oxide and baseline conditions and discrepancies in head motion quantified by average frame-wise displacements (n=16). FD: frame-wise displacement, GN+: global network of activation, GN-: global network of deactivation, VAN+: ventral attention network, VIS+: visual network, SMN+: somatomotor network, FPN+: frontoparietal network, DAN+: dorsal attention network, DMN+: default-mode network, p(B): Bonferroni–corrected p.

Table S3: ANCOVA results for principle gradient values with frame-wise displacement as a covariate

	Cases	Sum of Squares	df	Mean Square	F	p	p(B)
**SMN**	Drug	0.002	1	0.002	27.853	1.173e -5	8.211e -5
	FD	4.678e -6	1	4.678e -6	0.064	0.802	1
	Residual	0.002	29	7.275e -5			
**VIS**	Drug	4.496e -4	1	4.496e -4	3.231	0.083	0.581
	FD	4.861e -4	1	4.861e -4	3.493	0.072	0.504
	Residual	0.004	29	1.392e -4			
**DAN**	Drug	4.026e -6	1	4.026e -6	0.025	0.876	1
	FD	1.005e -4	1	1.005e -4	0.621	0.437	1
	Residual	0.005	29	1.618e -4			
**VAN**	Drug	2.836e -7	1	2.836e -7	0.002	0.966	1
	FD	3.648e -5	1	3.648e -5	0.241	0.627	1
	Residual	0.004	29	1.515e -4			
**LIM**	Drug	8.812e -5	1	8.812e -5	0.254	0.618	1
	FD	0.001	1	0.001	4.157	0.051	0.357
	Residual	0.01	29	3.465e -4			
**FPN**	Drug	0.001	1	0.001	19.186	1.413e -4	9.891e -4
	FD	5.914e -5	1	5.914e -5	0.969	0.333	1
	Residual	0.002	29	6.102e -5			
**DMN**	Drug	3.942e -4	1	3.942e -4	2.262	0.143	1
	FD	4.945e -4	1	4.945e -4	2.838	0.103	0.721
	Residual	0.005	29	1.743e -4			

Type III Sum of Squares. ANCOVA analyses were performed, incorporating FD as a covariate, to investigate the drug effect of nitrous oxide (n=16) on gradient values against baseline condition (n=16). FD: frame-wise displacement, SMN: somatomotor network, VIS: visual network, DAN: dorsal attention network, VAN: ventral attention network, LIM: limbic network, FPN: frontoparietal network, DMN: default-mode network, p(B): Bonferroni–corrected p.

Table S4: ANCOVA results for co-activation pattern occurrence rates with frame-wise displacement as a covariate

	Cases	Sum of Squares	df	Mean Square	F	p	p(B)
**GN+**	Drug	0.021	1	0.021	10.13	0.003	0.024
	FD	0.01	1	0.01	4.718	0.038	0.304
	Residual	0.059	29	0.002			
**GN-**	Drug	1.755e -5	1	1.755e -5	0.007	0.932	1
	FD	0.011	1	0.011	4.691	0.039	0.312
	Residual	0.068	29	0.002			
**VAN+**	Drug	0.009	1	0.009	6.501	0.016	0.128
	FD	7.566e -4	1	7.566e -4	0.577	0.454	1
	Residual	0.038	29	0.001			
**VIS+**	Drug	0.007	1	0.007	5.198	0.03	0.24
	FD	8.441e -4	1	8.441e -4	0.651	0.426	1
	Residual	0.038	29	0.001			
**SMN+**	Drug	0.008	1	0.008	9.289	0.005	0.04
	FD	0.011	1	0.011	11.599	0.002	0.016
	Residual	0.026	29	9.107e -4			
**FPN+**	Drug	0.008	1	0.008	11.941	0.002	0.016
	FD	0.004	1	0.004	5.77	0.023	0.184
	Residual	0.02	29	6.748e -4			
**DAN+**	Drug	0.004	1	0.004	1.567	0.221	1
	FD	1.710e -4	1	1.710e -4	0.066	0.799	1
	Residual	0.075	29	0.003			
**DMN+**	Drug	0.006	1	0.006	4.184	0.05	0.4
	FD	7.108e -4	1	7.108e -4	0.496	0.487	1
	Residual	0.042	29	0.001			

Type III Sum of Squares. ANCOVA analyses were performed, incorporating FD as a covariate, to investigate the drug effect of nitrous oxide (n=16) on CAP occurrence rates against baseline condition (n=16). FD: frame-wise displacement, GN+: global network of activation, GN-: global network of deactivation, VAN+: ventral attention network, VIS+: visual network, SMN+: somatomotor network, FPN+: frontoparietal network, DAN+: dorsal attention network, DMN+: default-mode network, p(B): Bonferroni–corrected p.

Table S5: Correlations between gradient values, CAP occurrence rates and 11D-ASC scores

Spearman Correlations

		EU	SE	BS	I	D	IC	A	CI	EI	AV	CMP	TOTAL
**CG_SMN**	rho	0.716	0.548	0.674	0.715	0.796	0.534	0.504	0.659	0.526	0.751	0.72	0.599
	p	3.9e -5	0.004	1.6e -4	4.0e -5	1.2e -6	0.005	0.009	2.5e -4	0.006	1.0e -5	3.4e -5	0.002
**CG_VIS**	rho	-0.014	0.167	0.059	-0.019	0.287	0.315	0.278	0.138	0.061	-0.028	0.149	0.215
	p	0.946	0.415	0.773	0.926	0.155	0.118	0.169	0.503	0.767	0.891	0.468	0.29
**CG_DAN**	rho	0.054	0.301	-0.058	-0.138	-0.027	-0.082	-0.096	-0.325	-0.024	-0.143	-0.135	-0.274
	p	0.793	0.136	0.777	0.5	0.895	0.69	0.641	0.105	0.908	0.487	0.511	0.175
**CG_VAN**	rho	0.031	-0.179	-0.029	-0.014	0.165	-0.018	-0.013	0.09	0.009	0.216	0.164	-0.059
	p	0.879	0.382	0.889	0.944	0.422	0.931	0.95	0.663	0.967	0.289	0.424	0.774
**CG_LIM**	rho	-0.048	0.104	-0.09	-0.034	0.093	0.037	0.053	-0.08	-0.016	-0.085	-0.036	0.015
	p	0.816	0.614	0.664	0.87	0.65	0.856	0.798	0.697	0.937	0.679	0.862	0.944
**CG_FPN**	rho	-0.389	-0.405	-0.322	-0.492	-0.635	-0.445	-0.326	-0.482	-0.217	-0.478	-0.5	-0.554
	p	0.05	0.04	0.109	0.011	4.9e -4	0.023	0.104	0.013	0.287	0.013	0.009	0.004
**CG_DMN**	rho	-0.197	-0.41	-0.164	-0.12	-0.468	-0.343	-0.371	-0.079	-0.217	-0.227	-0.331	-0.238
	p	0.334	0.038	0.423	0.56	0.016	0.087	0.062	0.703	0.288	0.264	0.099	0.24
**CAP_GN+**	rho	0.565	0.426	0.388	0.588	0.561	0.235	0.325	0.448	0.635	0.584	0.475	0.341
	p	0.003	0.03	0.05	0.002	0.003	0.248	0.105	0.022	4.9e -4	0.002	0.014	0.088
**CAP_GN-**	rho	0.155	0.251	-0.057	0.328	0.224	0.12	0.392	0.209	0.188	0.349	0.208	-0.017
	p	0.451	0.217	0.782	0.102	0.271	0.559	0.047	0.307	0.358	0.08	0.307	0.934
**CAP_VAN+**	rho	-0.013	0.065	0.047	-0.027	0.164	0.008	-0.178	0.189	-0.076	-0.104	-0.018	0.119
	p	0.948	0.752	0.821	0.894	0.422	0.97	0.384	0.356	0.713	0.615	0.931	0.561
**CAP_VIS+**	rho	0.075	0.289	0.183	0.125	0.283	0.271	0.18	0.298	0.101	0.027	0.101	0.283
	p	0.716	0.152	0.37	0.542	0.161	0.181	0.378	0.14	0.624	0.895	0.625	0.161
**CAP_SMN+**	rho	-0.169	-0.049	-0.308	-0.368	-0.355	-0.338	-0.123	-0.49	-0.175	-0.314	-0.337	-0.517
	p	0.41	0.81	0.126	0.065	0.075	0.091	0.548	0.011	0.393	0.118	0.092	0.008
**CAP_FPN+**	rho	-0.589	-0.356	-0.454	-0.49	-0.55	-0.147	-0.23	-0.584	-0.518	-0.463	-0.473	-0.527
	p	0.002	0.074	0.02	0.011	0.004	0.475	0.259	0.002	0.007	0.017	0.015	0.006
**CAP_DAN+**	rho	-0.103	-0.452	0.048	-0.125	-0.247	-0.043	-0.308	-0.032	-0.182	-0.147	-0.064	0.008
	p	0.616	0.02	0.817	0.543	0.224	0.834	0.126	0.875	0.373	0.474	0.757	0.971
**CAP_DMN+**	rho	-0.143	-0.357	-0.097	-0.355	-0.403	-0.289	-0.352	-0.284	-0.19	-0.275	-0.182	-0.086
	p	0.487	0.073	0.637	0.075	0.041	0.153	0.078	0.159	0.353	0.174	0.375	0.673

Spearman correlations were performed between gradient values, CAP occurrence rates and 11D-ASC scores across 13 participants and two conditions (n=26). CAP: co-activation pattern, CG: cortical gradient, SMN/ SMN+: somatomotor network, VIS/ VIS+: visual network, DAN/ DAN+: dorsal attention network, VAN/ VAN+: ventral attention network, LIM: limbic network, FPN/ FPN+: frontoparietal network, DMN/ DMN+: default-mode network, GN+: global network of activation, GN-: global network of deactivation, EU: experience of unity, SE: spiritual experience, BS: blissful state, I: insightfulness, D: disembodiment, IC: impaired control and cognition, A: anxiety, CI: complex imagery, EI: elementary imagery, AV: audio-visual synesthesia, CMP: changed meaning of percepts.
