# Supplementary material for: Psychedelic concentrations of nitrous oxide reduce functional differentiation in frontoparietal and somatomotor cortical networks
Source: Commun Biol. 2023 Dec 19;6:1284. doi: 10.1038/s42003-023-05678-1 (PMC10730842; doi:10.1038/s42003-023-05678-1)
Supplement: Supplementary file 4 — Reporting Summary [file 42003_2023_5678_MOESM4_ESM.pdf]

nature portfolio

Corresponding author(s): ZIRUI HUANG

Last updated by author(s): Nov 20, 2023

Reporting Summary

Nature Portfolio wishes to improve the reproducibility of the work that we publish. This form provides structure for consistency and transparency in reporting. For further information on Nature Portfolio policies, see our Editorial Policies and the Editorial Policy Checklist.

Statistics

For all statistical analyses, confirm that the following items are present in the figure legend, table legend, main text, or Methods section.

n/a	Confirmed	
☐	☒	The exact sample size (*n*) for each experimental group/condition, given as a discrete number and unit of measurement
☐	☒	A statement on whether measurements were taken from distinct samples or whether the same sample was measured repeatedly
☐	☒	The statistical test(s) used AND whether they are one- or two-sided<br>*Only common tests should be described solely by name; describe more complex techniques in the Methods section.*
☐	☒	A description of all covariates tested
☐	☒	A description of any assumptions or corrections, such as tests of normality and adjustment for multiple comparisons
☐	☒	A full description of the statistical parameters including central tendency (e.g. means) or other basic estimates (e.g. regression coefficient) AND variation (e.g. standard deviation) or associated estimates of uncertainty (e.g. confidence intervals)
☐	☒	For null hypothesis testing, the test statistic (e.g. *F*, *t*, *r*) with confidence intervals, effect sizes, degrees of freedom and *P* value noted<br>*Give P values as exact values whenever suitable.*
☒	☐	For Bayesian analysis, information on the choice of priors and Markov chain Monte Carlo settings
☒	☐	For hierarchical and complex designs, identification of the appropriate level for tests and full reporting of outcomes
☐	☒	Estimates of effect sizes (e.g. Cohen's *d*, Pearson's *r*), indicating how they were calculated

Our web collection on statistics for biologists contains articles on many of the points above.

Software and code

Policy information about availability of computer code

Data collection	Imaging data were obtained using a 3T Philips Achieva MRI scanner (Best, Netherlands) located at Michigan Medicine, University of Michigan.
Data analysis	Publicly available software and toolbox used for analyses include AFNI (http://afni.nimh.nih.gov/), MATLAB R2022a, BrainSpace (https://brainspace.readthedocs.io/en/latest/), and JASP v0.16.3 (https://jasp-stats.org/).

For manuscripts utilizing custom algorithms or software that are central to the research but not yet described in published literature, software must be made available to editors and reviewers. We strongly encourage code deposition in a community repository (e.g. GitHub). See the Nature Portfolio guidelines for submitting code & software for further information.

Data

Policy information about availability of data

All manuscripts must include a data availability statement. This statement should provide the following information, where applicable:

- Accession codes, unique identifiers, or web links for publicly available datasets
- A description of any restrictions on data availability
- For clinical datasets or third party data, please ensure that the statement adheres to our policy

All data needed to evaluate the conclusions in this article are present in the main text and the Supplementary Materials. Access to additional data by qualified investigators (i.e., affiliated with accredited academic and research institutions) are subject to scientific and ethical review. Completion of a material transfer agreement signed by an institutional official will be required in order to access the data.

Research involving human participants, their data, or biological material

Policy information about studies with human participants or human data. See also policy information about sex, gender (identity/presentation), and sexual orientation and race, ethnicity and racism.

Reporting on sex and gender	This study included 16 healthy participants (8 males, means ± SD, ages: 24.6±3.7 years).
Reporting on race, ethnicity, or other socially relevant groupings	Efforts were made to include male, female and minority enrollments in proportion to their presence in the local population. The Ann Arbor area consists of 74.7% white, 8.8% Black or African American, and 11.9% Asian, 0.3% American Indian and Alaska native, 0.1% Native Hawaiian or Pacific Islander residents (AreaConnect Ann Arbor Michigan Population and Demographics resource). Hispanic or Latino population is 3.4%. Advertising in a wide variety of metropolitan and community newspapers enabled us to acquire a sample that is representative of the local population and avoid recruiting biases on a racial basis.
Population characteristics	This study included 16 healthy participants (8 males, means ± SD, ages: 24.6±3.7 years). All participants were classified as American Society of Anesthesiologists (ASA) physical status I.
Recruitment	Requirements for participation, included being classified as physical status I by the American Society of Anesthesiologists, being free of drug abuse or psychosis, and being free of other health-related conditions (https://www.clinicaltrials.gov/ct2/show/NCT03435055).
Ethics oversight	The study was performed at the University of Michigan Medical School and received approval from the Institutional Review Board under the identifier HUM00096321.

Note that full information on the approval of the study protocol must also be provided in the manuscript.

Field-specific reporting

Please select the one below that is the best fit for your research. If you are not sure, read the appropriate sections before making your selection.

☒ Life sciences ☐ Behavioural & social sciences ☐ Ecological, evolutionary & environmental sciences

For a reference copy of the document with all sections, see nature.com/documents/nr-reporting-summary-flat.pdf

Life sciences study design

All studies must disclose on these points even when the disclosure is negative.

Sample size	This study included 18 healthy participants.
Data exclusions	Two participants were excluded because of excessive head motion (more than a half TRs in each scan).
Replication	Findings were replicated with and without the covariate of frame-wise displacement (FD).
Randomization	The experiments was not randomized because within-subject design was used.
Blinding	The investigators were not blinded to allocation during experiments and outcome assessment.

Reporting for specific materials, systems and methods

We require information from authors about some types of materials, experimental systems and methods used in many studies. Here, indicate whether each material, system or method listed is relevant to your study. If you are not sure if a list item applies to your research, read the appropriate section before selecting a response.

Materials & experimental systems

n/a	Involved in the study	
☒	☐	Antibodies
☒	☐	Eukaryotic cell lines
☒	☐	Palaeontology and archaeology
☒	☐	Animals and other organisms
☒	☐	Clinical data
☒	☐	Dual use research of concern
☒	☐	Plants

Methods

n/a	Involved in the study	
☒	☐	ChIP-seq
☒	☐	Flow cytometry
☐	☒	MRI-based neuroimaging

Plants

Seed stocks	n/a
Novel plant genotypes	n/a
Authentication	n/a

Magnetic resonance imaging

Experimental design

Design type	Resting state
Design specifications	6 minutes pre-nitrous oxide resting state and 6 minutes during nitrous oxide resting state.
Behavioral performance measures	Participants rated their experiences using altered-states-of-consciousness questionnaire.

Acquisition

Imaging type(s)	Functional
Field strength	3T
Sequence & imaging parameters	Functional whole-brain images were acquired using a T2*-weighted echo-planar sequence with the following parameters: 48 slices, TR/TE = 2000/30ms, slice thickness = 3 mm, field of view = 200 × 200mm, flip angle = 90°, and scan time of 6 minutes. High-resolution anatomical images were also acquired for co-registration with the resting state fMRI data.
Area of acquisition	Whole brain

Diffusion MRI ☐ Used ☒ Not used

Preprocessing

Preprocessing software	Standardized methods using AFNI (http://afni.nimh.nih.gov/).
Normalization	AFNI function: @auto_tlrc; a script to transform an antomical dataset to align with standard space template.
Normalization template	Talaraich stereotactic space (TT_N27).
Noise and artifact removal	Using AFNI's function 3dTproject, the time-censored data were band-pass filtered to 0.01 – 0.1 Hz. At the same time, various undesired components (e.g., physiological estimates, motion parameters) were removed via linear regression. The undesired components included linear and nonlinear drift, time series of head motion and its temporal derivative, and mean time series from the white matter and cerebrospinal fluid.
Volume censoring	Frame-wise displacement (FD) of head motion was calculated using frame-wise Euclidean Norm (square root of the sum squares) of the six-dimension motion derivatives. A frame and its each previous frame were excluded if the given frame's derivative value has a Euclidean Norm above FD=0.4 mm.

Statistical modeling & inference

Model type and settings	Cortical gradient analysis and co-activation patterns analysis.
Effect(s) tested	For network-based gradient analysis (mean gradient values) and CAP analysis, paired t-tests were conducted comparing the nitrous oxide condition (n=16) to the baseline (n=16). The Spearman correlations were used to analyze the relationship between the altered states questionnaire total scores and gradient values, as well as between the altered states questionnaire total scores and CAP occurrence rates, across 13 participants and two conditions (n=26). Bonferroni correction, i.e., dividing the critical P value α=0.05 by the number of comparisons being made, was used to counteract the multiple comparisons problem.

Specify type of analysis: ☐ Whole brain ☐ ROI-based ☒ Both

Anatomical location(s)	Seven-network parcellation scheme (Yeo et al., 2011)
Statistic type for inference (See Eklund et al. 2016)	Family-wise error rate of $p < 0.05$ with a minimum cluster size of 60 voxels.
Correction	Monte Carlo simulation was used via the AFNI program 3dClustSim.

Models & analysis

n/a	Involved in the study
☐	☒ Functional and/or effective connectivity
☒	☐ Graph analysis
☒	☐ Multivariate modeling or predictive analysis

Functional and/or effective connectivity	Pearson correlation
